# On the Use of Salix Nigra (Aments), a New Sexual Sedative, in the Treatment of Masturbation, Excessive Venery Spermatorrhœa, and Ovarian Disease

**Published:** 1886-02-20

**Authors:** F. T. Paine

**Affiliations:** Comanche, Texas


### ON THE USE OF SALIX NIGRA (AMENTS), A NEW SEXUAL SEDATIVE, IN THE TREATMENT OF MASTURBATION, EXCESSIVE VENERY SPERMATORRHŒA, AND OVARIAN DISEASE.

BY F. T. PAINE, M.D., COMANCHE, TEX.

[Read before the Texas Medical Association.]

In bringing before you for your consideration a new drug, I do so after mature reflection and a careful study of its necessity, and of its actual value as a therapeutic agent, in an active practical use of it for five years.

There has, perhaps, never been a time in the history of man when he did not need a sexual sedative, an agent capable of suppressing venereal desires, which, by indulgence, lead to excessive use of the sexual organs ; physiological in moderation, pathological in excess; leading to pathological states of the organs of both sexes, and also of the whole nervous system. No more pitiable object is presented to the professional eye than a neurasthenic youth of either sex spell-bound by a habit they have no power to break, when they have nothing better in their armamentarium than moral suasion, good advice, etc.

Just such cases set me to studying, years ago, to find something to set the captives free—to suppress the desire and the capacity at the same time. This I hapily found in the fluid extract salix nigra (aments or buds), or floral buds of the common willow tree of our Southern rivers, creeks, and lakes.

The first opportunity I had of trying the new remedy was in the case of Mr. McC——, who applied time and again from undefinable symptoms referable mostly to the genito-urinary organs, with a general neurasthenic state of the system. For weeks we failed to diagnose the case, until asked after his sexual passions and indulgences, when he candidly confessed that his desires had never been, and could not be satisfied—that he copulated six times every night, and still was unsatisfied ; and that his wife was in a bad state of health, and wished to see me. A pitiable state of mind and body ; a wife and two children to support, a crop planted and unable to work or do anything except gratify his passion, which, like the horse leech, whose demand was more, still more.

I had only four ounces of fluid extract salix nigra, the first sample I had ever seen. I handed it to him, with directions to take a teaspoonful three times a day, and to be sure to report in ten days, but not sooner. I counted up his copulations to amount to forty-two per week of seven nights. At the time appointed he came with his report. It was this : "I can hardly go to my wife one time a week now." You may be sure I was ready to exclaim, "Eureka ! Eureka !"

This occurred in 1880. Since then I have always had the medicine in reach, and have not failed to use it whenever an opportunity offered. Without details—which would weary you—I will state succinctly the outlines of a few cases :

One young man, aged twenty-six, in a decline of health, sold his land, became insane, and was taken by his friends to another county. In a correspondence with me he confessed to masturbating. I sent him a prescription of salix nigra, on using which he was profuse in his gratitude for relief from his bondage, and of his improved health. He is now in fine health.

Another, known as an inveterate masturbator, had lost his reason, and his physicians consulted with regard to the propriety of his castration as a *dernier resort*. On referring to us for advice, I prescribed salix nigra, and in due time one of his physicians reported suppression of the habit, suspension of the seminal losses, improvement of mind and general health, and that the salix was all that I claimed for it.

The next was a stranger here—a young man, twenty-four years old—a complete wreck. On application to me, I told him of his habits and the consequences, but I found he was married four years; that his wife had miscarried twice. She was 200 miles away. He got salix nigra, which stopped his emissions in less than ten days, and he soon sent for his wife, despite of my warnings. But by toning him up he has continued to improve, and now works for his living.

A lady, thirty-two years old; married thirteen years; sterile; very stout and robust; sought treatment for what she supposed a uterine disease. On investigation, I found hyperæsthesia of the ovaria, and gave her four ounces of fluid extract of salix nigra, with injunctions to report in ten days, which she did to this effect: "If a woman takes that medicine, she don't care if there is not a man in the world." I failed to get from this patient any expression as to her sexual passions previous to her treatment, but recently she informed me that the marital act had previously given her great pain—which was most probably the cause of her application for relief; doubtless a case of *dispareunia*.

Mrs. B—— had from puberty suffered from dysmenorrhœa, for fifteen or twenty years. She came under our care in 1882; aged thirty-five; sterile; suffering intensely one or two days every monthly period. I diagnosed the case endometritis, and supposed the endometrium the seat, the *fons et origio*, of all the trouble, and sought no further for a cause, until finding, to my great satisfaction, the endometrium very kindly healing. I was ready to congratulate all parties at the next catamenia, when, instead of congratulations, the dysmenorrhœa was in no wise relieved, and I had to stand by her bedside another night with morphia, chloral, chloroform, etc., to keep my patient alive or from acting the maniac. I then saw my mistake, and feeling for the ovaria, before neglected, I found them prolapsed and hyperæsthetic in the highest degree. I then reasoned that an agent so powerful to suppress passions dependent wholly on the functions of the physiological ovaria, must, as a matter of course, exercise a powerful influence in suppressing the pains of the pathological ovaria. I prescribed at once salix nigra in 1-drachm doses, three times a day. The next month the catamenia passed as pleasant as a May day, and so continued which will be two years in May, 1885. This lady, and husband, being very reticent, I never obtained any expression as to her sexual feelings before treatment, only that the medicine produced no change in her feelings.

The two following cases were sisters, treated in March and May, 1884; both bed-ridden for months with supposed uterine disease; had suffered all things from many physicians; and had vulva abscesses and vaginitis, etc. While at her bedside, and before prescribing, her husband informed me that his wife had never had any sexual passion. On examining, I found the ovaria hyperæsthetic, and prescribed salix nigra, in conjunction with necessary local treatment. The husband of the other made the same statement of want of passion, and the ovaria was likewise hyperæsthetic. She got salix nigra fluid extract (aments) June 1, 1884, and from having been bed-ridden 27th of May, when I saw her, she was in my office in Comanche on the 5th of July, thirty miles from home. At that visit she stated to me that, unless I could cure her pains, she wished me to remove the ovaria. She climbed into her wagon like a ten-year old girl, and went her way. I heard nothing more from her until September, 1884. Her father said he had not seen her since she was at my office in July, but that she had moved 150 miles, was doing her own housework, and had gained thirty pounds in weight. She wrote me in February, 1885, that her medicine was exhausted, and some of her troubles were returning.

Were I to write the half of the cases I have treated with the salix nigra your patience would be exhausted. The possibilities of the agent are very great—its future exceedingly promising. Before closing, I must say that, having used it five years, I have as much confidence in its virtues as you or I have in calomel or quinine in appropriate cases.

I have been careful in the cases cited to speak of them as hyperæsthetic, not as ovaritis. I have only found one case of ovaritis, or in which I could detect the products of inflammatory action. I have found in nearly every instance the left ovarium diseased. I am now treating a case of epilepsy of reflex ovarian origin, and she has wonderfully improved on the addition, to her use of the bromides, that of salix nigra.

In all the cases of ovarian disease I have found, in which I could obtain the state of sexual propensity, there was nearly or absolutely none. Oophorectomy was performed lately at Paducah, Ky. (see Medical and Surgical Reporter, March, 1884), by Dr.——— and others, for nymphomania, in which he reports no pathological appearance in excised organ. Salix nigra might have saved this woman her ovaria, had it been used.—*Southern Clinic.*

